# White matter abnormalities in amyotrophic lateral sclerosis: a free water imaging study

**DOI:** 10.3389/fnins.2026.1899073

**Published:** 2026-07-30

**Authors:** Zelin Liu, Haiqing Yang, Jiawei Cui, Zhengkun Guan, Duo Gao, Pingyong Feng, He Yang, Wenyi Li, Yuanhui Zhao, Qianhang Yang, Xuyang Zheng, Qi Liu, Zuojun Geng

**Affiliations:** 1 Department of Radiology, The Second Hospital of Hebei Medical University, Shijiazhuang, Hebei, China; 2 Department of Traditional and Western Medical Hepatology, Hebei Provincial Key Laboratory of Liver Fibrosis in Chronic Liver Diseases, Third Hospital of Hebei Medical University, Shijiazhuang, Hebei, China; 3 Department of Cardiology, The Fourth Hospital of Hebei Medical University, Shijiazhuang, Hebei, China; 4 Affiliated Hospital of Hebei University, Baoding, Hebei, China; 5 Department of Neurology, The Second Hospital of Hebei Medical University, Shijiazhuang, Hebei, China; 6 Hebei Key Laboratory of Medical Imaging, Shijiazhuang, China

**Keywords:** ALSFRS-R, amyotrophic lateral sclerosis, FW-DTI, MRI, TBSS

## Abstract

**Objective:**

To investigate white matter microstructural alterations in amyotrophic lateral sclerosis (ALS) using free-water-corrected diffusion tensor imaging (FW-DTI), compare its findings with those of conventional DTI, and examine the clinical correlations and preliminary diagnostic value of these metrics.

**Methods:**

44 ALS patients and 42 healthy controls underwent multi-b-value diffusion MRI. Conventional DTI metrics (fractional anisotropy [FA], mean diffusivity [MD], axial diffusivity [AxD], radial diffusivity [RD]), free-water-corrected metrics (FW-FA, FW-MD, FW-AxD, FW-RD), and the free-water fraction (FWF) were calculated. Tract-based spatial statistics (TBSS) was used for voxelwise group comparisons. Correlations between clinical parameters, including disease progression rate (ΔFS) and the Amyotrophic Lateral Sclerosis Functional Rating Scale-Revised (ALSFRS-R) score, and DTI metrics were examined. A diagnostic nomogram was constructed using logistic regression based on imaging markers that showed significant differences between groups.

**Results:**

Conventional DTI identified white matter abnormalities in ALS-related regions, including corticospinal tract-related regions, the corpus callosum, and the cingulate gyrus. FW-DTI showed additional and partially distinct alterations, including changes in the fornix, bilateral superior corona radiata, anterior and posterior corona radiata, and the posterior limb of the internal capsule. The free-water fraction did not differ between groups. Correlation analysis revealed that ΔFS was negatively associated with FA in the left posterior limb of the internal capsule (r = −0.432), and the ALSFRS-R score was positively associated with FW-FA in the right anterior corona radiata (r = 0.389). A diagnostic nomogram combining FA in the right cerebral peduncle and FW-FA in the right anterior corona radiata showed preliminary discriminative performance (area under the curve [AUC] = 0.860).

**Conclusion:**

FW-DTI may provide complementary model-derived information for characterizing ALS-related white matter alterations beyond conventional DTI. Specific regional metrics were associated with ΔFS and the ALSFRS-R score, and the preliminary diagnostic nomogram yielded an AUC of 0.860.

## 1 Introduction

Amyotrophic lateral sclerosis (ALS) is a severe neurodegenerative disorder characterized by the progressive degeneration of both upper and lower motor neurons, leading to limb and bulbar dysfunction and significantly reduced life expectancy (Feldman et al., 2022). Early diagnosis is limited by the heterogeneity of clinical manifestations and the lack of definitive biomarkers, often resulting in substantial diagnostic delays (Goutman et al., 2022a; Ilieva et al., 2023). As the disease progresses, patients develop limb weakness, impaired speech and swallowing, and ultimately respiratory failure (Goutman et al., 2022b).

Conventional magnetic resonance imaging (MRI) for ALS mainly reveals structural changes, but these changes are often subtle. Patients with ALS-D (ALS with dementia), a recognized clinical subtype of ALS with concurrent frontotemporal dementia, may present with frontal and temporal lobe atrophy and hyperintense signals in the subcortical white matter of both temporal lobes (Sato et al., 2009). However, these findings lack specificity and are insufficient for early diagnosis or disease progression monitoring. Diffusion tensor imaging (DTI) enhances the detection of white matter microstructural changes by measuring water diffusion directional coherence (fractional anisotropy, FA) and magnitude (mean diffusivity, MD). Multiple studies have consistently demonstrated decreased FA and increased MD in the corticospinal tract (CST) of ALS patients, providing imaging evidence of axonal damage and demyelination (Bao et al., 2018; Yin et al., 2008). Despite its promise, DTI faces clinical challenges; one study found no significant DTI parameter changes within six months (Alruwaili et al., 2019), possibly because substantial white matter damage has already occurred by symptom onset, suggesting the need for earlier detection or complementary imaging approaches. Beyond the corticospinal tract, conventional DTI studies have also reported extra-motor white matter abnormalities, including involvement of the corpus callosum and cingulate-related regions, suggesting that ALS-related white matter degeneration is not limited to the motor system (Zhang et al., 2018).

Free water (FW) imaging has emerged as a diffusion MRI technique for quantifying unbound extracellular water molecules (Botta et al., 2025). Previous studies have reported altered FW-related measures in neurodegenerative diseases such as Alzheimer’s disease (AD) and Parkinson’s disease (PD) (Xu et al., 2025; Kuang et al., 2025), whereas FW changes may differ across other neurological conditions, including multiple sclerosis (MS) and neuromyelitis optica spectrum disease (NMOSD) (Kim et al., 2024). These findings suggest that FW alterations may be disease- and context-dependent.

Importantly, conventional DTI assumes a single Gaussian diffusion process within each voxel (Ricchi et al., 2025), and its derived indices may be confounded when voxels contain both tissue and free water, such as CSF or edema-related extracellular water. In such cases, FA and MD may reflect mixed tissue and free-water signals rather than tissue microstructure alone (Hoy et al., 2014; Metzler-Baddeley et al., 2012). Given that ALS involves neuroinflammation and blood–CNS barrier impairment, this confounding effect may be relevant when interpreting diffusion abnormalities in this disease (Garbuzova-Davis and Sanberg, 2014). The bi-tensor FW-DTI model addresses this issue by modeling the diffusion signal as two compartments: an anisotropic tissue compartment and an isotropic free-water compartment, thereby estimating the free-water fraction and generating free-water-corrected tissue diffusion metrics (Pasternak et al., 2009). Although FW-DTI has been applied in other neurodegenerative diseases, including AD (Bergamino et al., 2021) and PD (Ofori et al., 2015), ALS-specific applications remain limited; recent ALS-related work has used free-water-corrected FA to assess the transcallosal motor pathway in ALS, but systematic whole-brain comparisons between conventional DTI and FW-DTI in ALS remain insufficiently characterized (Lehto et al., 2024).

Therefore, this exploratory study aimed to compare conventional DTI and FW-DTI in characterizing white matter abnormalities in ALS and to evaluate whether free-water correction reveals additional or spatially distinct alterations beyond conventional DTI. Clinical correlation and diagnostic performance analyses were conducted as preliminary, hypothesis-generating assessments rather than as validation of established clinical biomarkers.

## 2 Materials and methods

### 2.1 Study population

Patients diagnosed with ALS according to the revised El Escorial criteria (Airlie House criteria; 1998) (Brooks et al., 2000) were consecutively enrolled from the Second Affiliated Hospital of Hebei Medical University in Shijiazhuang, China, between March 2024 and November 2025. Patients and healthy controls (HCs) were matched for age, sex, and body mass index (BMI). Healthy controls were recruited from the same clinical center. The two groups showed no significant differences in age, sex, or BMI. Healthy controls were selected based on two key criteria: (1) absence of neurological abnormalities as confirmed by standardized neurological examination, and (2) no visible pathological changes on routine brain MRI scans. Ethical approval for this study was granted by the Ethics Review Committee of the Second Hospital of Hebei Medical University (Approval No. 2024-R193-N1), and all participants provided written informed consent prior to enrollment.

### 2.2 Clinical parameters of ALS

Clinical features of ALS were assessed using the ALSFRS-R score and the disease progression rate derived from ALSFRS-R. The ALSFRS-R evaluates functional domains including bulbar function, limb motor performance, and respiratory capacity, enabling assessment of functional status in ALS patients. The progression rate was calculated as: (48 − ALSFRS-R score)/disease duration (in months), where disease duration was defined as the time from symptom onset to the date of MRI scanning (Rooney et al., 2017).

### 2.3 MRI acquisition parameters

All participants underwent MRI scanning using a GE 3.0 T system equipped with a 48-channel head coil. Diffusion tensor imaging (DTI) data were collected with the following parameters: TR/TE = 4000/100.2 ms, flip angle = 90°, FOV = 224 × 224 mm^2^, matrix = 128 × 128, slice thickness = 3 mm, and voxel size = 1.8 × 1.8 × 3 mm^3^. Three b0 images and 31 diffusion-weighted images were acquired across three nonzero b-value shells of 650, 1,000, and 1,650 s/mm^2^, with 10, 11, and 10 non-collinear diffusion directions, respectively, enabling subsequent FW-DTI analysis.

The multi-shell protocol was designed to enable FW-DTI (Pasternak et al., 2009), for which previous optimization work has suggested that inclusion of a relatively high b-value shell around 1,500 s/mm^2^ may improve estimation of the fast isotropic diffusion component while maintaining clinical feasibility (Hoy et al., 2014). Accordingly, the highest shell in the present study was set to 1,650 s/mm^2^ as a pragmatic compromise between free-water separation, signal-to-noise ratio achievable with the 48-channel head coil, and acquisition time tolerability in ALS patients, who may present with bulbar or respiratory dysfunction. We acknowledge that this b-value is higher than that commonly used in conventional DTI acquisitions and may increase the contribution of non-Gaussian diffusion effects, potentially influencing tensor estimation and the absolute values of conventional DTI-derived metrics (Jones and Basser, 2004). Although all participants were scanned and processed using the same protocol, reducing the likelihood of group-specific acquisition bias, this methodological issue should still be considered when interpreting conventional DTI metrics. Therefore, the conventional DTI findings should be interpreted with appropriate caution, whereas the multi-shell acquisition was primarily optimized for FW-DTI estimation.

### 2.4 Image processing

DTI data were preprocessed using FSL (FMRIB Software Library; https://fsl.fmrib.ox.ac.uk/fsl/fslwiki/). Key procedures involved: (1) isolating the b0 image, (2) carrying out brain extraction via BET (Smith, 2002), and (3) correcting for head motion and eddy current-induced distortions by aligning all diffusion-weighted images to the b = 0 s/mm^2^ reference image using an affine transformation (Andersson and Sotiropoulos, 2015), with the gradient directions adjusted accordingly using FSL (Jenkinson et al., 2012). After preprocessing, diffusion tensors were fitted using a least-squares approach implemented in dtifit, generating fractional anisotropy (FA), mean diffusivity (MD), axial diffusivity (AxD), and radial diffusivity (RD) maps for each subject.

In the conventional DTI analysis, the diffusion-weighted signal within each voxel is fitted using a single-compartment tensor model, and the derived diffusion metrics therefore reflect the combined contribution of all water compartments within that voxel. In contrast, the FW-DTI model employs a bi-tensor representation that separates the extracellular isotropic free-water component from the tissue-specific anisotropic diffusion signal (Pasternak et al., 2009). For each diffusion-weighted measurement, the normalized signal attenuation is modeled as:


$$S(b,g)/S_{o}=(1-f)\cdot \exp(-b\cdot g^{T}D\_\text{tissue}\;g)+f\cdot \exp(-b\cdot D\_\text{free}),$$

where f is the free-water volume fraction (FWF), D_tissue is the tissue diffusion tensor, g is the diffusion gradient direction, and D_free is fixed to 3 × 10^−3^ mm^2^/s, the diffusivity of free water at body temperature (Hoy et al., 2014).

In this framework, the FW map reflects the estimated extracellular free-water contribution, whereas free-water-corrected FA, MD, AxD, and RD are derived from the tissue tensor after accounting for the isotropic free-water compartment. Thus, FW-DTI differs from conventional DTI by reducing free-water contamination and providing model-derived tissue-compartment diffusion metrics.

Critically, fitting this bi-tensor model to single-shell data is mathematically ill-posed without additional constraints and prone to estimation errors (Correia et al., 2024). To improve model estimation and reduce this limitation, we employed a multi-shell diffusion acquisition protocol. Free-water (FW) maps, along with free-water-corrected FA (FW-FA) and MD (FW-MD) maps, were computed using a bi-tensor model implemented in DIPY (Python) (Hoy et al., 2014; Pasternak et al., 2009). From the same model, free-water-corrected AxD (FW-AxD) and RD (FW-RD) maps were also derived.

### 2.5 Tract-based spatial statistics (TBSS)

Tract-based spatial statistics (TBSS) (Smith et al., 2006) was used to investigate white matter microstructural properties based on diffusion MRI metrics, including fractional anisotropy (FA), mean diffusivity (MD), axial diffusivity (AxD), radial diffusivity (RD), free water (FW), and their free-water-corrected versions: FW-FA, FW-MD, FW-AxD, and FW-RD. Given the limited prior evidence regarding FW-DTI alterations in ALS, the whole-brain TBSS analysis was designed as an exploratory analysis rather than a confirmatory region-specific hypothesis test.

Briefly, individual FA maps were first linearly aligned to the FMRIB58 template (1 × 1 × 1 mm^3^) and then nonlinearly registered to the FMRIB58_FA template in MNI space using the standard TBSS pipeline. A group-averaged FA image was created and skeletonized using an FA threshold of 0.2 to restrict the analysis to the core white matter skeleton, consistent with standard TBSS practice. The same nonlinear transformation parameters derived from the FA registration were applied to project all other diffusion metrics (MD, AxD, RD, FW, FW-FA, FW-MD, FW-AxD, FW-RD) onto the mean FA skeleton.

Voxelwise group comparisons were performed using a general linear model within FSL’s randomize framework (Winkler et al., 2014), with age and sex included as covariates. Statistical inference was based on non-parametric permutation testing (5,000 permutations). To control for multiple comparisons, voxel-wise family-wise error (FWE) correction was applied using the --vox option in randomize, which directly assesses significance at each voxel based on the null distribution of the maximum statistic generated by permutation (Nichols and Holmes, 2002). Voxels with FWE-corrected *p* < 0.05 were considered statistically significant. Significant regions were identified using the JHU ICBM-DTI-81 white matter atlas (Oishi et al., 2008) within FSL.

### 2.6 ROI-based analysis

Based on the statistically significant clusters identified from the exploratory whole-brain TBSS analysis, subsequent ROI-based extraction was performed. For each atlas-defined region, the mean values of the diffusion parameters estimated by conventional DTI and FW-DTI were calculated for each subject. This ROI-based analysis was conducted as a *post hoc* exploratory analysis to summarize the magnitude of diffusion abnormalities within atlas-defined regions and to support subsequent preliminary clinical correlation and modeling analyses. To ensure measurement reliability and minimize the influence of very small clusters, only ROIs containing more than 20 contiguous voxels were retained. This minimum cluster-size criterion was used as a pragmatic threshold to reduce instability from very small voxel clusters and was not intended as an additional voxel-wise significance threshold. Although these regions had already survived whole-brain FWE correction, we additionally applied Bonferroni correction across the extracted ROIs in subsequent correlation and modeling analyses. Because these ROIs were derived from the same whole-brain group comparison, subsequent ROI-level analyses were interpreted as post hoc exploratory summaries rather than independent confirmatory tests.

### 2.7 Statistical analysis

Voxel-wise statistical analysis was performed using TBSS with family-wise error (FWE) correction (*p* < 0.05). Significant clusters were then localized to regions in the Johns Hopkins University (JHU) white matter atlas. From these regions, three ROIs—the FA of the right cerebral peduncle, the FA of the left posterior limb of the internal capsule (L-PLIC), and the FW-FA of the right anterior corona radiata—were selected.

To account for multiple comparisons, Bonferroni correction was applied to these three variables (significance threshold: *p* < 0.017). All three variables remained statistically significant: FA of the right cerebral peduncle (*p* = 0.001), FA of the left posterior limb of the internal capsule (*p* = 0.001), and FW-FA of the right anterior corona radiata (*p* = 0.007). A logistic regression model was used to construct a nomogram. Internal validation was performed using the bootstrap method. Calibration curves were used to assess the agreement between predicted and observed probabilities, and the area under the ROC curve (AUC) was used to evaluate the model’s discriminative ability.

Statistical analyses were performed using R (version 4.4.1) and IBM SPSS Statistics (version 27.0). A two-tailed *p* < 0.05 was considered statistically significant, except where the Bonferroni-corrected threshold applied.

## 3 Results

### 3.1 Participants’ demographics and clinical assessment

**Figure 1:**
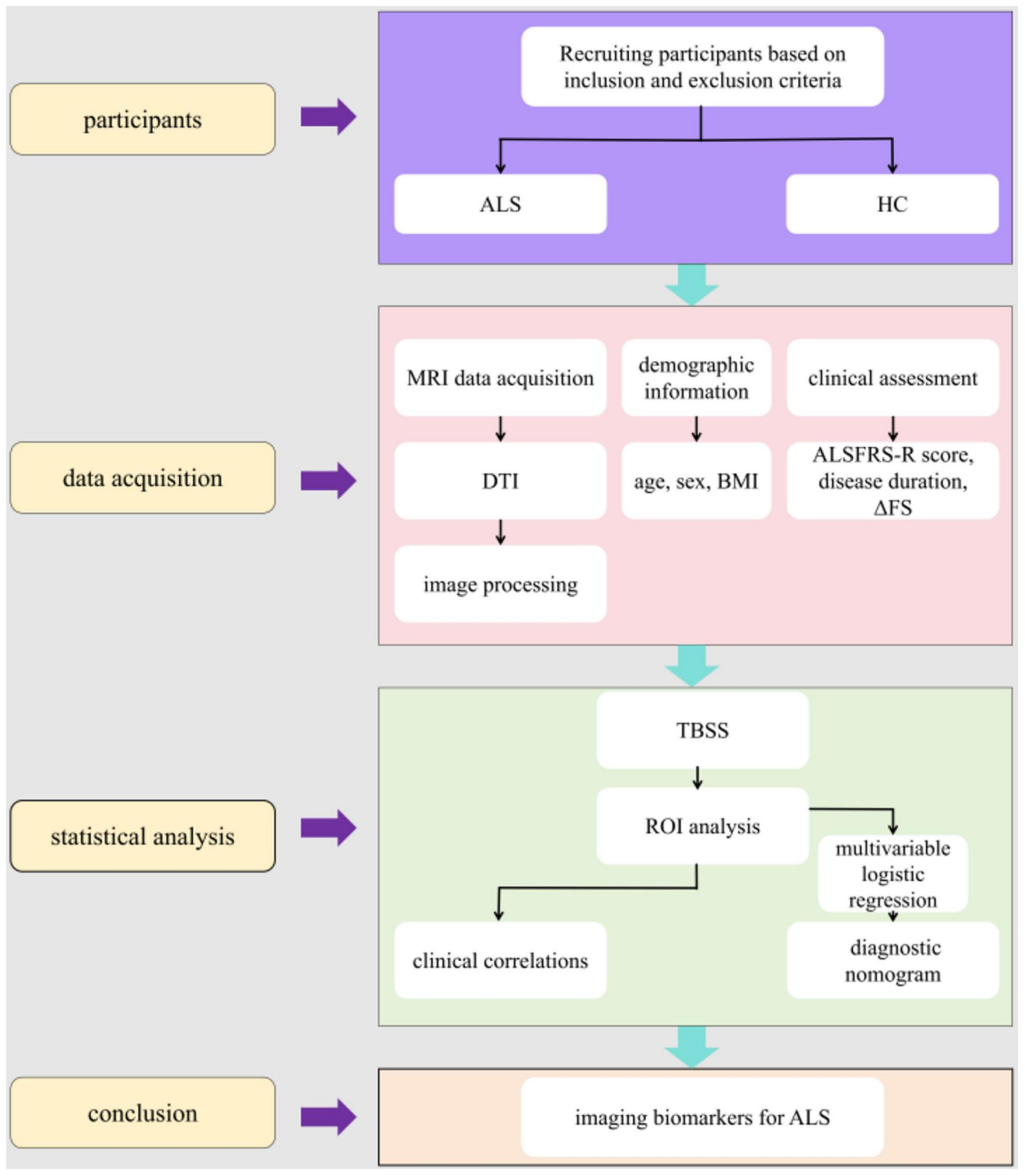
Study’s flow diagram.

Figure 1 displays the study’s flow diagram. A total of 86 participants were enrolled in this study, including 44 patients with ALS and 42 healthy controls. Baseline demographic characteristics and clinical data are summarized in Table 1. There were no significant differences between the ALS and HC groups in age, sex distribution, body mass index (BMI), handedness, or years of education (all *p* > 0.05). Among patients with ALS, 34 had spinal-onset disease and 10 had bulbar-onset disease. The mean ALSFRS-R score was 36.5 ± 5.8, the mean progression rate was 0.8 ± 0.4 per month, and the median disease duration was 13.5 months (interquartile range, 10–24 months).

**Table 1:** Baseline characteristics of participants.

	ALS (*n* = 44)	HC (*n* = 42)	*p*-value
Age	57.6 ± 9.4	56.2 ± 5.6	0.413
**Sex**			0.875
Female	15	15	
Male	29	27	
BMI	23.5 ± 3.0	23.8 ± 2.5	0.685
Right-handed	44	42	> 0.999
Education (years)	9 (6, 12)	12 (9, 12)	0.216
**Site of onset**			
Spinal (limb)	34		
Bulbar	10		
ALSFRS-R score	36.5 ± 5.8		
Progression rate (/month)	0.8 ± 0.4		
Disease duration (months)	13.5 (10, 24)		

Data are presented as mean ± standard deviation or median (interquartile range), as appropriate. ALS, amyotrophic lateral sclerosis; ALSFRS-R, revised amyotrophic lateral sclerosis functional rating scale; HC, healthy controls; BMI, body mass index.

### 3.2 White matter skeleton comparisons: patients vs. controls

**Figure 2:**
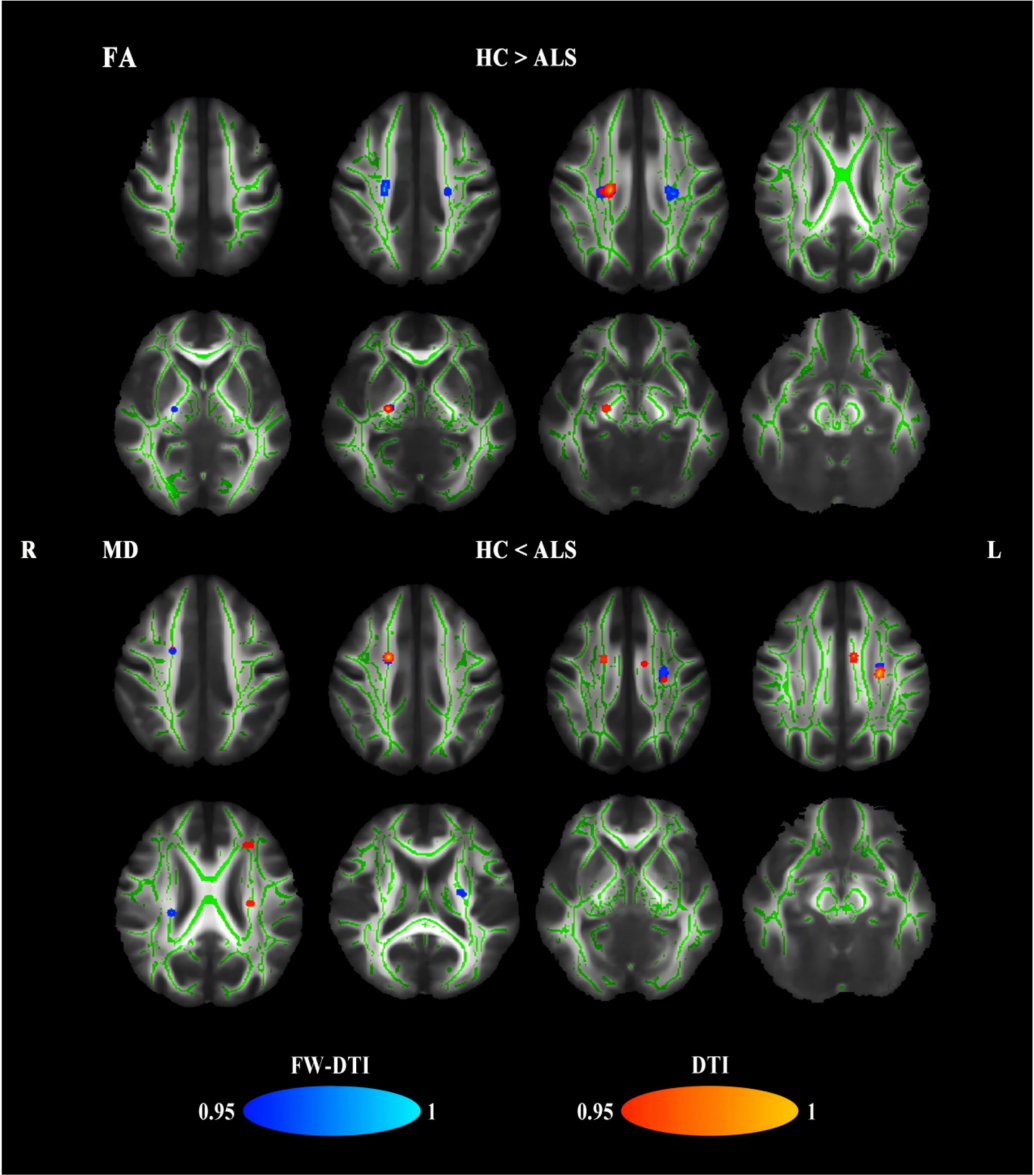
Locations of statistically significant differences in white matter tracts for FA (top) and MD (bottom). These parametric maps display regions with increased values in control subjects compared with patients for both conventional DTI (red) and FW-DTI (blue). Grayscale: FA white matter skeleton map (range: 0–1). The red–yellow scale (range: 0.95–1) represents the (1 − p) values for DTI parameters, while the blue–light blue scale (range: 0.95–1) represents the (1 − p) values for FW-DTI parameters. R = right; L = left.

**Figure 3:**
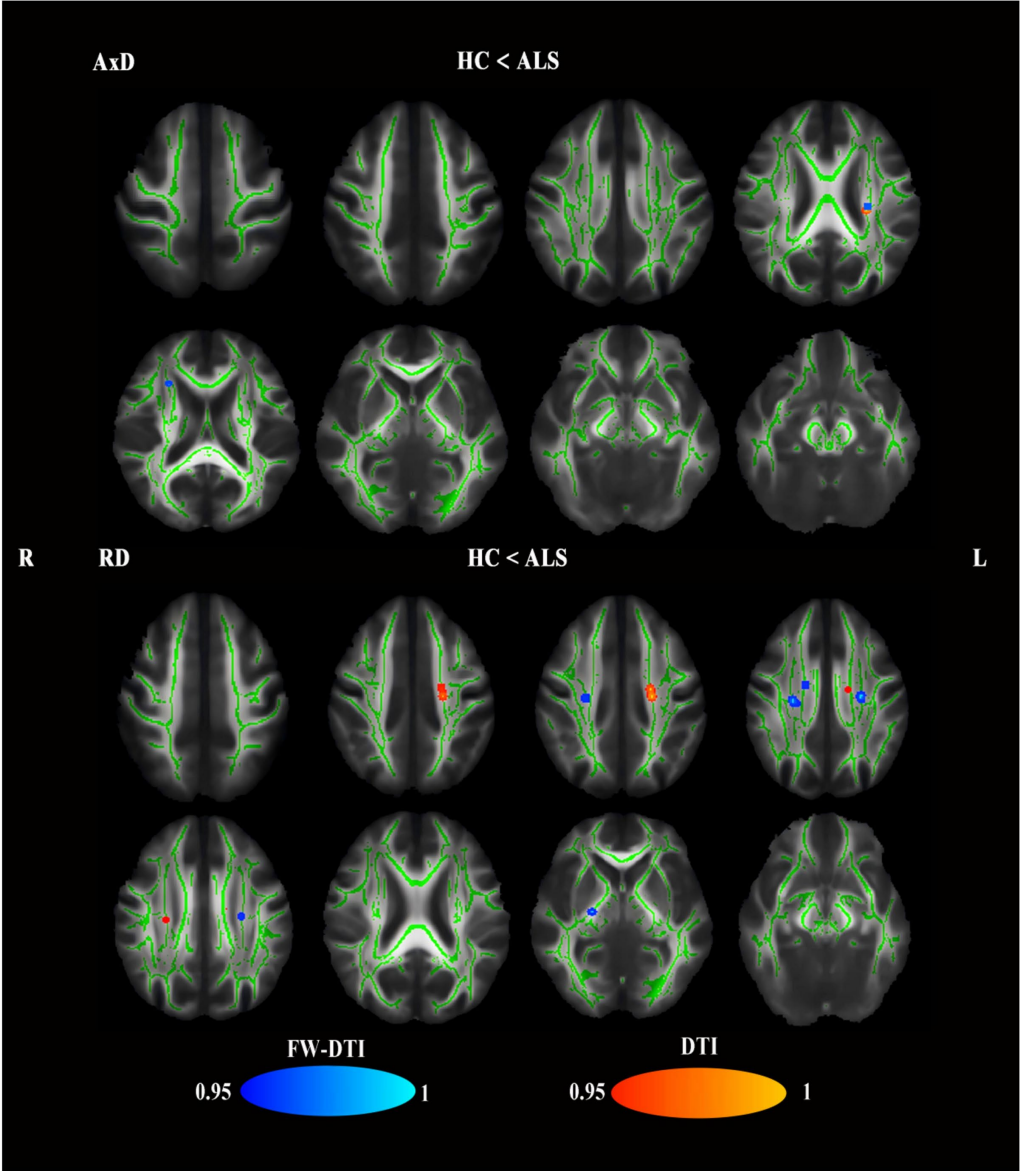
Locations of statistically significant differences in white matter tracts for AxD (top) and RD (bottom). These parametric maps display regions with increased values in control subjects compared with patients with ALS for both conventional DTI (red) and FW-DTI (blue). Grayscale: FA white matter skeleton map (range: 0–1). The red–yellow scale (range: 0.95–1) represents the (1 − p) values *for DTI p*arameters, while the blue–light blue scale (range: 0.95–1) represents the (1 − p) values for FW-DTI parameters. R = right; L = left.

The tract-based spatial statistics (TBSS) analysis revealed significant differences in white matter diffusion metrics between ALS patients and healthy controls, with all reported clusters surviving voxel-level correction for multiple comparisons (*p* < 0.05). Anatomical labels were assigned according to the JHU-ICBM-DTI-81 white matter atlas. Overall, FW-DTI revealed additional and partially distinct white matter diffusion abnormalities compared with conventional DTI across diffusion metrics, as detailed in Tables 2, 3 and illustrated in Figures 2, 3. No significant group differences were observed in the free-water fraction (FWF).

**Table 2:** Significant white matter regions for DTI-FA, FW-FA DTI-MD, and FW-MD metrics.

	Voxels	*p*-value	MNI			Voxels	*p*-value	MNI		
			X	Y	Z			X	Y	Z
**JHU-ICBM-DTI-81 WM Atlas**	**DTI-FA**					**FW-FA**				
Fornix (column and body of fornix)						1	0.04	2	-4	9
Cerebral peduncle R	37	0.001	20	−14	−5					
Anterior corona radiata R						48	0.007	20	−17	40
Superior corona radiata R	2	0.04	20	−17	39	5	0.004	25	−18	37
Superior corona radiata L						1	0.04	−22	−20	38
Posterior limb of internal capsule R						2	0.012	20	−14	−2
Posterior limb of internal capsule L	20	0.001	−20	−15	−4					
**JHU-ICBM-DTI-81 WM atlas**	**DTI-MD**					**FW-MD**				
Anterior corona radiata L	1	0.024	−24	27	23					
Superior corona radiata R	11	0.006	19	−1	41	3	0.001	19	−4	42
Superior corona radiata L	11	0.001	−26	−16	31	11	0.001	−26	−12	35
Posterior corona radiata R						2	0.029	25	−24	25
Posterior limb of internal capsule L						1	0.013	−23	−10	15
Cingulum (cingulate gyrus) L	2	0.023	−8	−2	33					
Body of corpus callosum	1	0.017	−11	−5	35					

All reported clusters survived correction for multiple comparisons at the voxel level (*p* < 0.05), with cluster sizes ≥1 voxel.

FA = fractional anisotropy; MD = mean diffusivity; DTI = diffusion tensor imaging; FW-DTI = free-water-corrected diffusion tensor imaging; FW-FA = free-water-corrected fractional anisotropy; FW-MD = free-water-corrected mean diffusivity; R = right; L = left. JHU-ICBM-DTI-81 WM atlas = Johns Hopkins University International Consortium for Brain Mapping DTI-81 white matter atlas.

**Table 3:** Significant white matter regions for DTI-AxD, FW-AxD, DTI-RD, and FW-RD metrics.

	Voxels	*p*-value	MNI			Voxels	*p*-value	MNI		
			X	Y	Z			X	Y	Z
**JHU-ICBM-DTI-81 WM Atlas**	**DTI-AxD**					**FW-AxD**				
Anterior corona radiata R						2	0.007	26	20	16
Superior corona radiata L	2	0.018	−25	−23	25	1	0.044	−26	−20	24
JHU-ICBM-DTI-81 WM atlas	DTI-RD					FW-RD				
Cerebral peduncle L	10	0.004	−19	−15	−5					
Anterior corona radiata R	6	0.021	26	−17	33					
Superior corona radiata R						16	0.001	25	−19	37
Superior corona radiata L	18	0.001	−20	−15	42	15	0.001	−25	−16	35
Posterior limb of internal capsule R						3	0.012	22	−14	0
Body of corpus callosum	1	0.042	−15	−11	34	2	0.04	16	−8	35

All reported clusters survived correction for multiple comparisons at the voxel level (*p* < 0.05), with cluster sizes ≥1 voxel.

AxD = axial diffusivity; RD = radial diffusivity; DTI = diffusion tensor imaging; FW-DTI = free-water-corrected diffusion tensor imaging; FW-AxD = free-water-corrected axial diffusivity; FW-RD = free-water-corrected radial diffusivity; R = right; L = left; JHU-ICBM-DTI-81 WM atlas = Johns Hopkins University International Consortium for Brain Mapping DTI-81 white matter atlas.

#### 3.2.1 Fractional anisotropy (FA) findings

For FA-derived metrics, conventional DTI showed significant reductions in ALS patients compared with healthy controls in the right cerebral peduncle (37 voxels, *p* = 0.001), the left posterior limb of the internal capsule (20 voxels, *p* = 0.001), and the right superior corona radiata (2 voxels, *p* = 0.040).

FW-DTI-derived FA also showed a significant reduction in the right superior corona radiata (5 voxels, *p* = 0.004), and additionally identified significant FA reductions in the fornix (1 voxel, *p* = 0.040), the right anterior corona radiata (48 voxels, *p* = 0.007), the left superior corona radiata (1 voxel, *p* = 0.040), and the right posterior limb of the internal capsule (2 voxels, *p* = 0.012).

#### 3.2.2 Mean diffusivity (MD) findings

For MD-derived metrics, conventional DTI showed significant increases in ALS patients compared with healthy controls in the left anterior corona radiata (1 voxel, *p* = 0.024), the right superior corona radiata (11 voxels, *p* = 0.006), the left superior corona radiata (11 voxels, *p* = 0.001), the body of the corpus callosum (1 voxel, *p* = 0.017), and the left cingulum/cingulate gyrus region (2 voxels, *p* = 0.023).

FW-DTI-derived MD also showed significant increases in the right superior corona radiata (3 voxels, p = 0.001) and the left superior corona radiata (11 voxels, p = 0.001), and additionally identified significant MD increases in the right posterior corona radiata (2 voxels, *p* = 0.029) and the left posterior limb of the internal capsule (1 voxel, *p* = 0.013).

#### 3.2.3 Axial diffusivity (AxD) and radial diffusivity (RD) findings

For AxD-derived metrics, conventional DTI showed a significant increase in ALS patients compared with healthy controls in the left superior corona radiata (2 voxels, *p* = 0.018).

FW-DTI-derived AxD also showed a significant increase in the left superior corona radiata (1 voxel, *p* = 0.044), and additionally identified a significant AxD increase in the right anterior corona radiata (2 voxels, *p* = 0.007).

For RD-derived metrics, conventional DTI showed significant increases in ALS patients compared with healthy controls in the left cerebral peduncle (10 voxels, *p* = 0.004), the right anterior corona radiata (6 voxels, *p* = 0.021), the left superior corona radiata (18 voxels, *p* = 0.001), and the body of the corpus callosum (1 voxel, *p* = 0.042).

FW-DTI-derived RD also showed significant increases in the left superior corona radiata (15 voxels, *p* = 0.001) and the body of the corpus callosum (2 voxels, *p* = 0.040), and additionally identified significant RD increases in the right superior corona radiata (16 voxels, *p* = 0.001) and the right posterior limb of the internal capsule (3 voxels, *p* = 0.012).

Collectively, these findings suggest that FW-DTI may provide complementary model-derived information relative to conventional DTI in this cohort.

### 3.3 ROI-based analysis results

In the ROI-based analysis, three ROIs met the inclusion criteria and showed significant group differences after Bonferroni correction (corrected threshold: *p* < 0.017): the right anterior corona radiata (R-ACR; FW-FA, *p* = 0.007), the right cerebral peduncle (R-CP; conventional FA, *p* = 0.001), and the left posterior limb of the internal capsule (L-PLIC; conventional FA, *p* = 0.001).

### 3.4 Exploratory cross-sectional correlations between clinical status and white matter microstructural integrity in ALS patients

We examined the relationships between clinical parameters—retrospectively calculated disease progression rate (ΔFS) and functional status (ALSFRS-R score)—and DTI-derived microstructural metrics (FA and FWFA) within the ROIs identified from the preceding TBSS and ROI-based analyses (Figure 3). This analysis was exploratory and cross-sectional, as MRI was acquired at a single time point. Therefore, ΔFS was used only as a clinical index of disease progression rate, rather than as evidence of longitudinal imaging change. After false discovery rate (FDR) correction for multiple comparisons, two significant correlations were observed.

First, disease progression rate (ΔFS) showed a significant negative correlation with fractional anisotropy (FA) in the left posterior limb of the internal capsule (L-PLIC) (r = −0.432, q = 0.0206), indicating that faster disease progression was associated with lower microstructural integrity in this tract.

Second, functional status (ALSFRS-R score) showed a significant positive correlation with free-water-corrected FA (FWFA) in the right anterior corona radiata (R-ACR) (r = 0.389, q = 0.0272), suggesting that higher functional ability was associated with better microstructural integrity in this region.

**Figure 4:**
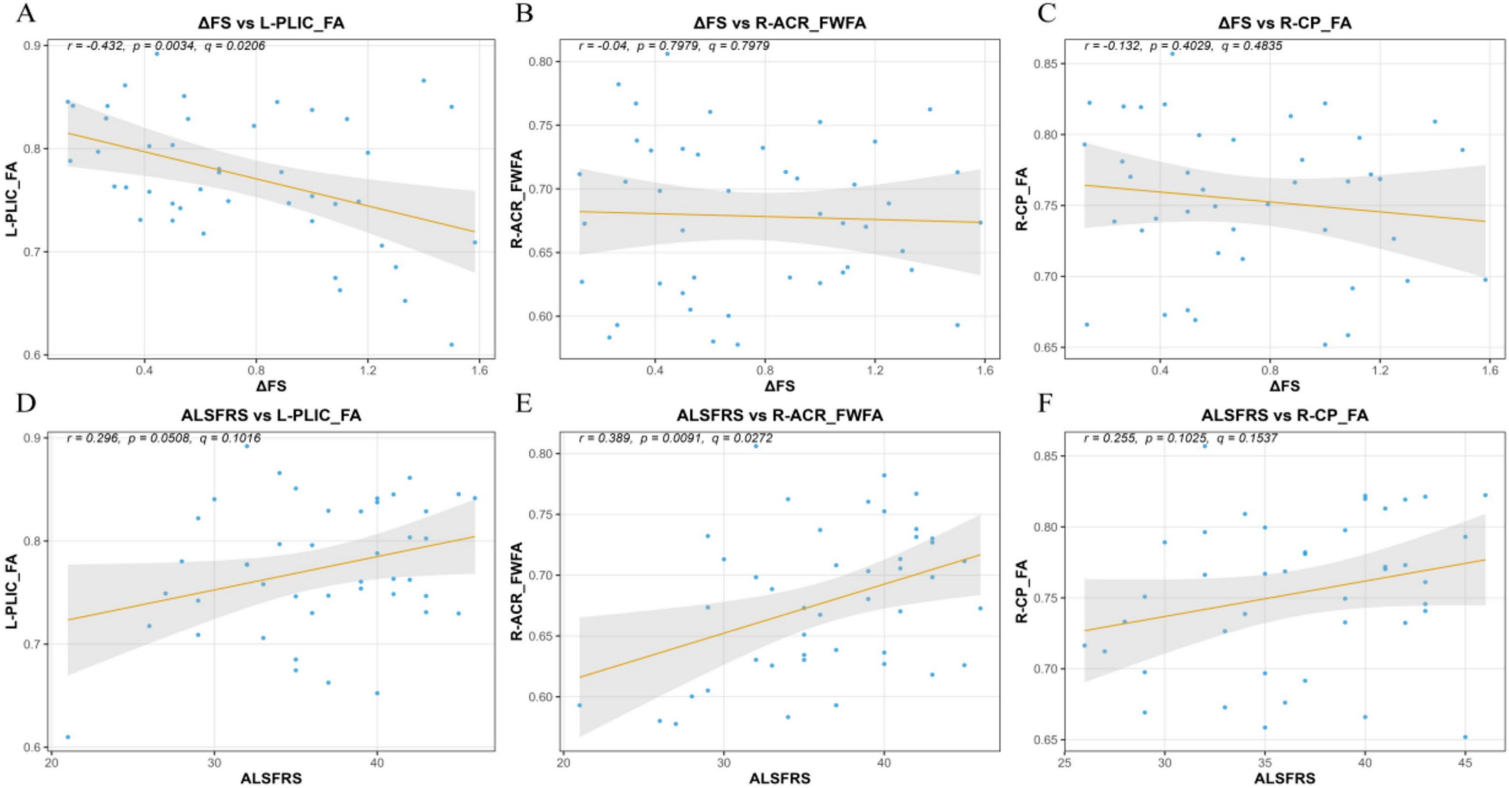
Scatter plots illustrating the relationships between clinical measures and white matter microstructural integrity. **(A–C)** Correlations between ΔFS and FA in the left posterior limb of the internal capsule (L-PLIC), FWFA in the right anterior corona radiata (R-ACR), and FA in the right cerebral peduncle (R-CP). **(D–F)** Correlations between the ALSFRS score and the same three diffusion metrics. Orange lines represent linear regression fits, and shaded gray areas denote the 95% confidence intervals of the regression. Pearson’s correlation coefficient (*r*), uncorrected *p*-value, and false discovery rate (FDR)-corrected *q*-value are provided for each analysis. Significant correlations (FDR-corrected *q* < 0.05) were observed between ΔFS and L-PLIC FA (A, *r* = −0.432, *q* = 0.0206) and between ALSFRS score and R-ACR FWFA (E, *r* = 0.389, *q* = 0.0272). Original figure labels use “ALSFRS” for brevity; this corresponds to the ALSFRS-R score used throughout the manuscript.

No other significant associations between clinical measures and DTI metrics were identified (all q > 0.05). In Figure 4, “ALSFRS” is used as an abbreviation for ALSFRS-R for simplicity.

### 3.5 Preliminary development and internal validation of a DTI-based diagnostic model for ALS

**Figure 5:**
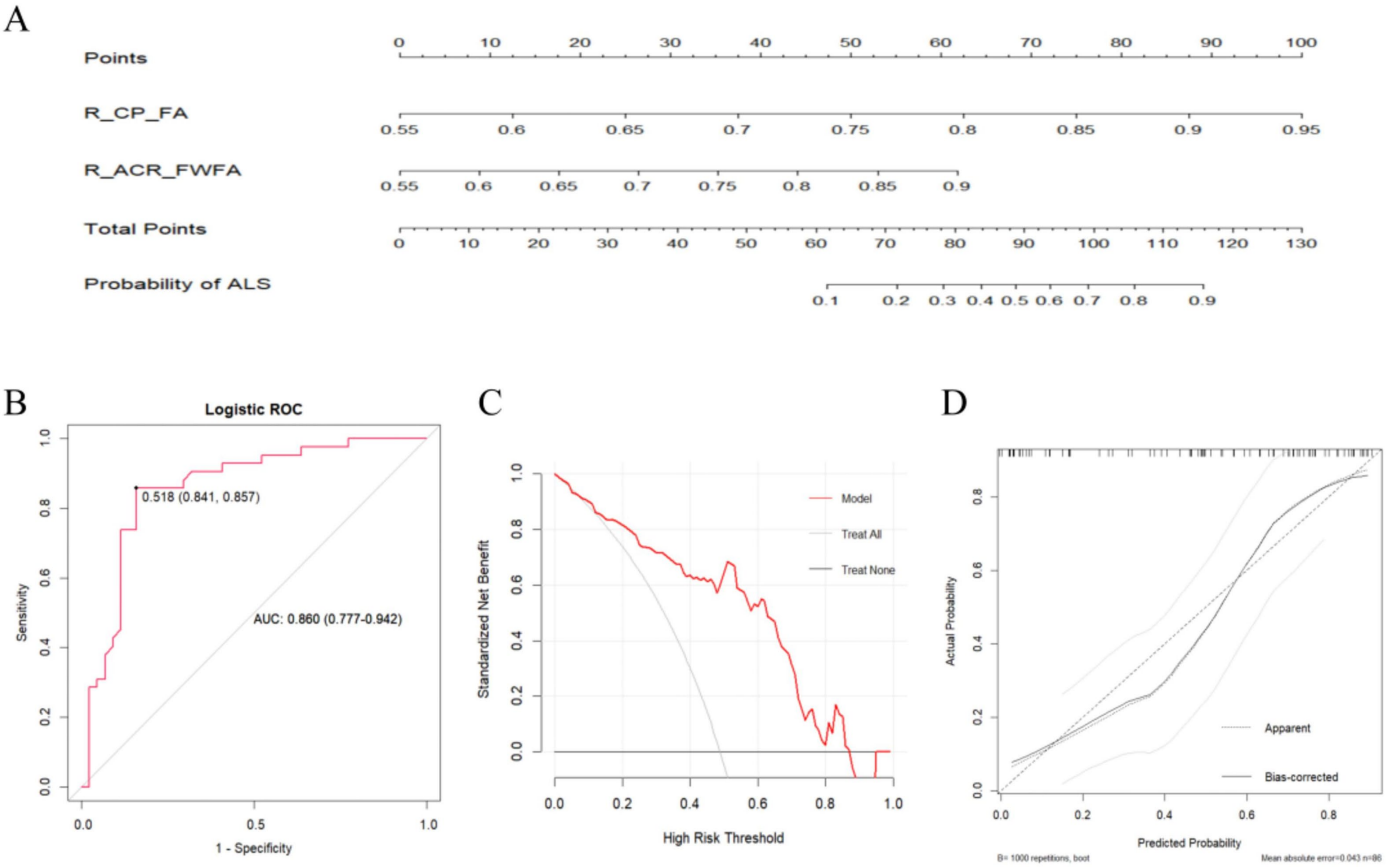
Diagnostic performance and internal validation of the combined diffusion tensor imaging-based prediction model for amyotrophic lateral sclerosis. **(A)** Nomogram constructed using two imaging metrics (R_CP_FA and R_ACR_FWFA) to predict the probability of ALS. **(B)** Receiver operating characteristic (ROC) curve of the model. **(C)** Decision curve analysis (DCA) showing the standardized net benefit of the prediction model across different high-risk thresholds. **(D)** Calibration curve assessing the agreement between predicted and actual probabilities of ALS.

Among the three ROIs showing group differences, multivariable logistic regression retained two imaging markers in the final model (R_CP_FA and R_ACR_FWFA) to construct a preliminary diagnostic nomogram for ALS (the FA of the left posterior limb of the internal capsule was not retained). The model showed apparent discriminative ability, with an AUC of 0.860. Decision curve analysis showed that, across the examined risk thresholds, the nomogram provided a higher standardized net benefit than the two extreme strategies, classifying all subjects as ALS or all as healthy controls. Internal calibration with 1,000 bootstrap repetitions showed good agreement between predicted and observed probabilities. Given the limited sample size and absence of an external validation cohort, this model should be interpreted as exploratory and requires independent validation before clinical application (Figure 5).

## 4 Discussion

This study combined FW-DTI with multi-b-value diffusion MRI and TBSS to assess free-water-corrected white matter alterations in ALS. Key findings include: (1) conventional DTI identified abnormalities in classical ALS-related white matter regions, including corticospinal tract (CST)-related regions, the corpus callosum, and the cingulate gyrus; and (2) FW-DTI identified additional white matter regions not detected by conventional DTI, including the fornix, the right posterior limb of the internal capsule, and the anterior/posterior corona radiata. These findings suggest that FW-DTI may provide complementary tissue-compartment information for characterizing white matter alterations in ALS. However, because the present study lacks histopathological confirmation, independent replication, or external validation, the detection of additional regions should not be interpreted as evidence of higher sensitivity or superiority over conventional DTI.

Conventional DTI parameters, including FA, MD, AxD, and RD, reflect different aspects of white matter microstructure (Tae et al., 2018). In this study, FA was decreased in the right cerebral peduncle, left posterior limb of the internal capsule, and right superior corona radiata—regions that constitute key segments of the corticospinal tract (CST)—consistent with previously reported ALS-related white matter changes associated with upper motor neuron degeneration, axonal loss, and demyelination (De Marchi et al., 2020). The increase in MD may reflect reduced microstructural integrity or increased extracellular diffusivity. AxD was abnormal only in the left superior corona radiata, whereas RD was abnormal in the left cerebral peduncle, right anterior corona radiata, left superior corona radiata, and body of the corpus callosum. These findings may indicate heterogeneous white matter microstructural alterations in ALS; however, specific pathological interpretations, such as axonal injury or demyelination, should be made cautiously because DTI-derived metrics are not pathologically specific (Yoshiura et al., 2002; Chondrogiorgi et al., 2019).

FW-DTI models a separate isotropic free-water compartment and may reduce the influence of extracellular free-water contamination. This approach may be useful in regions susceptible to partial volume effects. Previous studies have suggested that FW correction can reveal diffusion abnormalities that are not fully captured by conventional DTI (Guadilla et al., 2025). Therefore, the additional regions identified by FW-DTI in the present study should be interpreted as complementary model-derived findings rather than as definitive evidence of superior sensitivity.

The involvement of non-motor pathways, including the fornix, cingulate gyrus, and corpus callosum, is consistent with the concept that ALS may affect extra-motor networks in addition to motor pathways. However, because cognitive and behavioral assessments were not available in the present study, correlations between these imaging findings and extra-motor clinical manifestations could not be performed; therefore, these findings should be interpreted cautiously. The fornix is an important limbic white matter tract involved in hippocampal connectivity and has been associated with memory and executive function in previous studies (Srisaikaew et al., 2020; Hou et al., 2024). ALS-FTD spectrum studies have also reported fornix and limbic system abnormalities in patients with cognitive or behavioral impairment (Branco et al., 2018). Thus, the observed FW-FA reduction in the fornix may indicate involvement of limbic-related white matter pathways in ALS, but it cannot be directly linked to specific memory, emotional, or executive symptoms in the present cohort.

Although FA abnormalities were not detected in the cingulate gyrus or corpus callosum, we observed MD elevation in the left cingulate gyrus and RD elevation in the body of the corpus callosum. Previous studies in other neurodegenerative disorders have linked cingulate and corpus callosum microstructural abnormalities to cognitive decline or executive dysfunction (Zheng et al., 2014; Zhang et al., 2007). These findings may suggest extra-motor white matter involvement in ALS, but this interpretation remains exploratory in the absence of direct cognitive or behavioral correlations.

The additional regions identified by FW-DTI may be biologically relevant. The fornix is adjacent to the lateral ventricle and is susceptible to cerebrospinal-fluid partial volume effects (Metzler-Baddeley et al., 2012). The isolated FW-FA abnormality in the fornix may therefore reflect a tissue-compartment alteration that was less apparent on conventional DTI after accounting for free-water contamination. However, this finding should not be interpreted as direct evidence of hippocampal efferent fiber damage without pathological or longitudinal validation. Similarly, the anterior corona radiata may be relevant to frontal-subcortical pathways, but its functional implication remains indirect without cognitive testing. Findings from other neurodegenerative diseases provide useful context (Bergamino et al., 2021; Planetta et al., 2016), but extrapolation to ALS should be made cautiously.

In this study, no significant group difference was observed in the free-water fraction. This suggests that the additional FW-DTI findings were not simply driven by a global increase in extracellular free water. Nevertheless, FW-DTI metrics are model-derived estimates and should not be interpreted as direct evidence of histopathological damage. Future studies combining FW-DTI with multimodal imaging, longitudinal follow-up, and clinical assessments are needed to clarify their biological significance.

Three imaging metrics (R-ACR_FWFA, R-CP_FA, L-PLIC_FA) were retained from the *post hoc* ROI-based analysis because they showed significant group differences after correction for multiple comparisons and met the predefined minimum cluster-size criterion for ROI extraction.

Our clinical analyses further suggested potential associations between selected imaging metrics and disease features. ΔFS showed a significant negative correlation with FA in the left posterior limb of the internal capsule, suggesting a potential association between this region and disease progression (Menke et al., 2012). ALSFRS-R scores were positively correlated with FW-FA in the right anterior corona radiata, consistent with previous reports linking anterior corona radiata integrity to clinical scores (Zhang et al., 2018). However, these clinical correlations should be regarded as exploratory and require confirmation in larger cohorts.

Following logistic regression analysis, R-CP_FA and R-ACR_FWFA were incorporated into the nomogram. L-PLIC_FA correlated with disease progression but did not enter the final model, possibly suggesting that imaging markers related to disease progression and those contributing to diagnostic classification may not fully overlap. The combined nomogram achieved an AUC of 0.860 in the present cohort, indicating preliminary discriminative performance. However, because the model was only internally validated, its clinical utility remains exploratory and requires external validation.

This study has several limitations. First, the cross-sectional design and single-time-point MRI acquisition preclude causal or longitudinal interpretation of the relationship between white matter alterations and disease progression. Longitudinal studies with repeated MRI assessments are therefore needed. In addition, although the relatively high b-value of 1,650 s/mm^2^ was selected as a methodological trade-off for FW-DTI feasibility and clinical applicability, it may increase non-Gaussian diffusion effects and influence conventional DTI tensor estimation and absolute diffusion metrics. Therefore, the conventional DTI findings should be interpreted cautiously, as the acquisition protocol was primarily optimized for multi-shell FW-DTI rather than for a separate conventional single-shell DTI analysis. Second, cognitive and behavioral assessments, such as the Edinburgh Cognitive and Behavioral ALS Screen (ECAS) (Niven et al., 2015), were not available, limiting direct interpretation of white matter alterations in non-motor pathways. In addition, subgroup analyses according to site of onset were not performed because of the limited and imbalanced sample size, particularly the small number of bulbar-onset patients. Future studies incorporating dedicated cognitive and behavioral measures and larger clinically stratified cohorts may help clarify the clinical relevance of these findings. Third, the isotropic assumption of the free-water model may not fully hold in regions with complex fiber architecture, fiber crossing, or pathological extracellular water accumulation (Hoy et al., 2014). Therefore, FW-DTI-derived metrics should be interpreted as model-based estimates. SWI was not acquired in the present study, so correlations with SWI-related markers could not be assessed. Future multimodal, longitudinal, or pathological validation studies are needed. Fourth, because this was an exploratory imaging study based on available participants, no formal *a priori* power calculation was performed. The clinical correlation analyses and diagnostic nomogram should therefore be regarded as preliminary and hypothesis-generating. Moreover, the nomogram was internally validated only and lacked an independent external validation cohort, limiting its current diagnostic utility and generalizability. In addition, because disease control groups were not included, the specificity of FW-DTI for ALS relative to other neurological or neurodegenerative disorders could not be evaluated.

Finally, although L-PLIC_FA was associated with disease progression, it was not retained in the final nomogram. This may suggest that imaging markers related to disease progression and diagnostic classification do not fully overlap, but this interpretation remains preliminary and requires confirmation in larger independent cohorts. Furthermore, medication use and socioeconomic information were not systematically available and were therefore not included in the present analysis.

## 5 Conclusion

This exploratory study used FW-DTI, multi-b-value diffusion MRI, and TBSS to characterize white matter abnormalities in ALS. Compared with conventional DTI, FW-DTI showed additional and partially distinct model-derived diffusion alterations in the fornix, corona radiata, and posterior limb of the internal capsule, suggesting that free-water correction may provide complementary information for assessing ALS-related white matter involvement. FA in the left posterior limb of the internal capsule and FW-FA in the right anterior corona radiata were associated with clinical progression rate and ALSFRS-R score, respectively. The diagnostic nomogram showed preliminary discriminative performance; however, it requires validation in larger independent cohorts. These findings should not be interpreted as evidence of higher sensitivity without independent validation or pathological confirmation.

## Data Availability

The original contributions presented in the study are included in the article/supplementary material, further inquiries can be directed to the corresponding authors.
